# “Strong Teeth”—a study protocol for an early-phase feasibility trial of a complex oral health intervention delivered by dental teams to parents of young children

**DOI:** 10.1186/s40814-019-0483-9

**Published:** 2019-08-13

**Authors:** Kerina Tull, Kara A. Gray-Burrows, Amrit Bhatti, Jenny Owen, Lucy Rutter, Timothy Zoltie, Jayne Purdy, Erin Giles, Carron Paige, Morvin Patel, Zoe Marshman, Robert West, Sue Pavitt, Peter F. Day

**Affiliations:** 1 University of Leeds, Leeds Institute of Health Sciences, Clarendon Way, Leeds, LS2 9NL UK; 2 University of Leeds, School of Dentistry, Clarendon Way, Leeds, LS2 9LU UK; 3 University of Sheffield, School of Clinical Dentistry, Claremont Crescent, Sheffield, S10 2TA UK

**Keywords:** Complex intervention, Oral health behaviour change, General dental practice, Dental team, Decay, Parental supervised toothbrushing, Diet, Parents, Young children, Electric toothbrush

## Abstract

**Background:**

Dental attendance provides an important opportunity for dental teams to explore with parents the oral health behaviours they undertake for their young children (0–5 years old). For these discussions to be effective, dental professionals need to be skilled in behaviour change conversations. The current evidence suggests that dental teams need further support, training and resources in this area. Therefore, the University of Leeds and Oral-B (Procter & Gamble Company) have worked with the local community and dental professionals to co-develop “Strong Teeth” (an oral health intervention), which is delivered in a general dental practice setting by the whole dental team. The protocol for this early phase study will explore the feasibility and acceptability of the Strong Teeth intervention to parents and the dental team, as well as explore short-term changes in oral health behaviour.

**Methods:**

Forty parents (20 of children aged 0–2 years old, and 20 of children aged 3–5 years old) who are about to attend the dentist for their child’s regular dental check-up will be recruited to the study. Parents and children will be recruited from 4 to 8 different dental practices. In the home setting, consent and baseline oral health behaviour data will be collected. The researchers will ask parents questions about their child’s oral health behaviours, including toothbrushing and diet. Three different proxy objective measures of toothbrushing will be collected and compared with self-report measures of parental supervised toothbrushing (PSB).

**Discussion:**

The parent and child will then attend their dental visit and receive the Strong Teeth intervention, delivered by the dental team. This intervention should take 5–15 min to be delivered, in addition to the routine dental check-up. Furthermore, children aged 0–2 years old will receive an Oral-B manual children’s toothbrush, and children aged 3–5 years old will receive an Oral-B electric rechargeable children’s toothbrush. At 2 weeks and 2–3 months following the Strong Teeth intervention, further self-report and objective measures will be collected in the parent/child’s home. This data will be supplemented with purposively sampled qualitative interviews with parents (approximately 3 months following the intervention) and dental team members (following delivery of the intervention).

**Trial registration:**

ISRCTN Register, (ISRCTN10709150)

**Electronic supplementary material:**

The online version of this article (10.1186/s40814-019-0483-9) contains supplementary material, which is available to authorized users.

## Background

Dental caries (tooth decay) is the most prevalent preventable childhood disease and a major public health priority [1]. Caries is a disease of health inequality. In England, 12% of 3- and 23% of 5-year-olds are affected by caries, with figures rising to 17% and 40% for children living in deprived parts of Yorkshire, respectively [2].

Both Public Health England [3] and the National Institute for Health and Care Excellence [4] identify young children and their parents as a key focus for oral health advice. Supporting parents to initiate and adopt protective home-based oral health behaviours in early-life is critical to the development of long-term oral health habits, thereby reducing common oral diseases such as caries and periodontal disease across the life course [5–7]. Both dental teams and parents [8–10] have identified that changing poor oral health behaviours for children is challenging, especially once dental disease has already been identified. Therefore, an approach which is strongly supported by local communities [11] is to encourage good oral health behaviours from the outset with different early years professionals skilled in providing appropriate support and advice. Following the development of our generic complex oral health intervention [11], our research group have adapted the intervention for different health and early years professionals. One such example is the HABIT intervention, which is focused on the universal developmental review undertaken by health visitors [12]. This home visit with parents of children aged 9–12 months covers a wide range of general health topics including a short conversation around oral health. The HABIT intervention involves training of health visitors to improve the structure, content and quality of these oral health conversations, as well as providing supporting paper-based and digital resources.

During the development of the generic and HABIT interventions, the community and study participants have repeatedly identified the need for preventive oral health conversations delivered by the primary care dental team. However, nearly two thirds (65.9%) of 0–4-year-olds did not attend the dentist in the 12 months up to June 2018 [13] and hence the need for effective oral health conversations in both the dental and community settings. These dental attendance figures are a key driver for a national oral health initiative in England, Dental Check by One (DCby1), which aims to encourage parents to take their child to the dentist before their first birthday (https://dentalcheckbyone.co.uk/) and establish regular dental attendance behaviours. The frequency of attendance is determined by the dental team based on an oral health risk assessment and can vary between 3 and 12 months [14]. Although Dental Check by One is aimed at tackling non-attendance, attendance in itself does not necessarily mean prevention advice is provided or adopted. To maximise the benefits of dental attendance, dental teams need to be able to have effective behaviour change conversations. As an example, a recent randomised controlled trial undertaken in Northern Ireland showed over a third of children developed dental caries by the age of 6 years old, despite regular attendance at the dentist over the previous 3 years [15]. In this study, preventive advice followed national Public Health England guidelines [3]. This highlights that changing oral health behaviours is challenging and requires more than simply providing information to parents.

There have been several studies that focus on the experiences of dental teams in providing oral health advice to patients [16–20]. These have identified a number of challenges, including the “ad hoc” nature of the content and delivery of oral health advice, the lack of training, knowledge and personal skills, as well as pressures related to insufficient finances, staff, facilities and time. Whilst national guidelines [3] have clarified what oral health behaviours should be promoted, they do not identify how to effectively undertake these behaviour change conversations.

Oral health behaviours (for example, brushing teeth twice a day with a fluoride toothpaste and reducing the frequency and amount of sugar consumed) are complex as they are influenced at multiple levels (i.e. individual, interpersonal, community, organisational and environmental), which can act as both barriers and facilitators to adoption [11, 21]. As such, effective oral health interventions must embrace appropriate complex intervention (traditionally defined as interventions with several interacting components) methodology, underpinned by psychological theory, as outlined by the Medical Research Council [16]. This is the approach that has been taken when developing the “Strong Teeth” intervention, such that as well as providing the evidence-based guidance provided in “Delivering Better Oral Health”, there is a strong recognition and appreciation of the challenges families with young children face and how this can impact on caring for their children’s teeth, which is based on our previous research that is underpinned by the Theoretical Domains Framework and socio-ecological model [11, 21]. For example, despite lacking the capability to effectively brush their own teeth, many young children are responsible for their own toothbrushing, yet, children are not always engaged nor co-operative with parental involvement. This is one of the reasons why in the early-phase evaluation of the Strong Teeth intervention, we have included the provision of an electric toothbrush in the 3–5 year old, as the novelty of the brush may increase child engagement with toothbrushing and parental involvement. However, accessing the acceptability and impact of electric toothbrushes in terms of engagement, toothbrushing behaviours as well as other issues, such as cost and ease of use, will be essential in the present study to determine whether this forms a key component of the intervention. The key strength of the Strong Teeth intervention is the training and focus on the conversation between dental professional and parent. Utilising a whole team approach, the conversation is tailored to the needs of each family and encourages parents to identify their own challenges and subsequently, the solutions to overcome these challenges. Yet allows each conversation to be delivered with consistency and clarity due to its structured and hierarchical format.

In collaboration with Oral-B (Procter & Gamble Company), the University of Leeds has undertaken a programme of research to develop a complex oral health intervention, delivered by dental teams to parents of young children. This programme of work included undertaking a series of rapid reviews to identify (1) the barriers and facilitators to toothbrushing, and healthy eating in respect to oral health for children aged 0–11 years old; and (2) Interventions already developed for use in general dental practice and their efficacy in reducing dental caries. As we had previously qualitatively explored the experiences of parents of children aged 0 – 6 years old [21], a second workstream explored qualitatively the experiences of dental teams (*n* = 27), parents (*n* = 37) and children (aged 7–10 years old, involving five classes in three different schools) in delivering and receiving oral health advice and what impact this had on parents’ and children’s behaviour. This was to assess what the range and scope of the intervention should be (i.e. was a combined or separate approach needed for different age groups). This work led to the Strong Teeth intervention concentrating on the 0–5 year age group. Using our earlier generic complex intervention work [11, 17, 21] in conjunction with this research, we have worked with Oral-B to co-develop the Strong Teeth intervention (https://www.dentalcare.co.uk/en-gb/strong-teeth-strong-kids). As part of a co-production approach to development, 12 focus groups with dental professionals (*n* = 4, *k* = 27) and parents (*n* = 8, *k* = 41) were undertaken to review and incrementally improve the intervention. Full details of the rapid reviews, qualitative interviews and co-production process are not in the scope of the current paper and will be reported elsewhere. Nevertheless, the Strong Teeth intervention is now finalised and ready for an early-phase evaluation to explore its acceptability to parents and dental teams, the feasibility of delivery, and whether it leads to behaviour change.

## Aims/objectives

### Feasibility study primary aim

To undertake an early-phase feasibility trial of the Strong Teeth intervention delivered by dental teams to parents of children aged 0–5 years old.

### Feasibility study primary objectives

Using a mixed-methods approach (including self-report questionnaires, dental examinations, filming the toothbrushing interaction between parent and child, and qualitative interviews):

1. To explore with NHS dental teams, the acceptability and feasibility of delivering the Strong Teeth intervention to parents of children aged 0–5 years old
2. To review study findings against progression criteria (see Table 1) and determine whether progression to a definitive trial is appropriate

**Table 1:** Progression criteria to definitive trial, without remedial action taken to trial design

Adoption and maintenance of appropriate oral health behaviours at 2–3 month follow-up (≥ 80%) based on self-report measures		
Intervention mechanism produces intended changes in the determinants of oral health behaviour		
Process evaluation	a	Feasibility of delivering the “Strong Teeth” intervention in a dental setting
	b	Intervention, and self-reported and objective outcome measures are acceptable to dental teams and parents
	c	Adequate recruitment (≥ 25%) of eligible families for data collection
	d	Adequate retention (≥ 85%) of consented families to data completion

### Feasibility study secondary objectives

The secondary objectives are as follows:

1. To explore with parents of children aged 0–5 years old the acceptability of the Strong Teeth intervention
2. To study the mechanisms of action for the Strong Teeth intervention
3. To correlate different proxy objective measures of toothbrushing with parental self-reports of parental supervised toothbrushing (PSB, i.e. the parent actively brushing their child’s teeth)
4. To describe the changes in dietary behaviour and PSB as a result of the Strong Teeth intervention in children aged 0–5 years old
5. To examine the impact of providing children aged 3–5 years old with an Oral-B electric rechargeable toothbrush, with respect to acceptability and toothbrushing behaviours

## Design/methods

This mixed-methods study will involve two participant groups: Group A—dental teams working in NHS dental practices (n = 4-8 practices) and Group B–parents of children aged 0–5 years old (*n* = 40) to allow the objectives to be achieved and to capture the perspectives of all relevant stakeholders. Involvement of participants from different backgrounds is essential to ensure the sample is representative of the local population. Therefore, this study will seek to involve parents from different socio-economic and ethnic minority groups.

### Overall design of the study

In parts of Yorkshire (Bradford, Leeds and surrounding areas) where many children are at high risk of dental caries, 40 parents who are about to attend the dentist for their child’s regular dental check-up (20 parents of children aged 0–2 years old, and 20 parents of children aged 3–5 years old) will be recruited from 4 to 8 different dental practices.

In the home setting, consent and baseline oral health behaviour data will be collected. The researcher will ask parents questions about their children’s oral health behaviours, including toothbrushing [18] and dietary behaviours [19] based on validated measures (the full baseline questionnaire can be found in Additional file 1: Appendix 1). Three different proxy objective measures of PSB will be collected and compared to self-reported parental behaviours: (1) children’s pre-brushing plaque levels per sextant [20]; (2) duration of toothbrushing and parent-child interaction during toothbrushing—the researcher will film the parent/child toothbrushing using a small action camera (GoPro HERO5, GoPro .Inc) and this will be subsequently evaluated by the research team using an established toothbrushing index, please see Additional file 1: Appendix 2 [22]; and (3) toothbrushing activity—parents will be provided with either a paper Magic Timer diary or Disney Magic Timer app for their phone/tablet, which records frequency and duration of toothbrushing. It is imperative to obtain objective as well as self-reported measures of toothbrushing as research has shown there tends to be a mis-match between reported and observed behaviours [23, 24]. The dental team member will also collect the gingivitis rating per sextant [25] and number of teeth present, missing and decayed following training and calibration using British Association for the Study of Community Dentistry (BASCD) standards [26, 27].

The parent and child will then attend their NHS dental check-up and receive the Strong Teeth intervention delivered by the dental team. The Strong Teeth resources, training manual and videos are targeted at the whole dental team to enable them to have effective oral care conversations with parents of young children in their practice. The Strong Teeth intervention serves to provide a structure and hierarchy to the conversation and can be roughly broken into three sections: (1) Check motivation—why is oral health important? (2) Check brushing technique—how to brush? (3) Identifying other barriers to oral health (e.g. healthy eating, influence of family and friends, managing the child’s behaviour to enable brushing, remembering to brush)—how to overcome these barriers? A variety of paper-based and digital resources for both dental professionals and parents are available to support the conversation (a full implementation guide, including the behaviour change techniques underlying the intervention and the Delivering Better Oral Health guidance covered by the intervention, is available from (https://www.dentalcare.co.uk/en-gb/strong-teeth-strong-kids).

Two weeks and 2–3 months following the Strong Teeth intervention, further self-reports of toothbrushing and dietary behaviours and objective measures of PSB will be collected in the parent/child’s home. This measurement schedule is shaped by the time taken for habitual behaviours to become established [28].

Recruitment and retention rates will be recorded, as this will be essential to establish the feasibility of undertaking a definitive trial (see Table 1 for the full progression criteria) The design for each group (Group A—NHS dental teams and Group B—parents of children aged 0–5 years old) will now be discussed in turn.

### Acceptability and feasibility to dental teams delivering the Strong Teeth intervention to parents of children 0-5 years old

#### Training

Each dental team member who will deliver the Strong Teeth intervention will attend a training session delivered by members of the research team (PD, KG-B, AB, JP, LR, JO and KT). The session will include evidence-based techniques for undertaking a behaviour change conversation and different approaches to engaging and motivating parents, including those who initially display resistance to behaviour change. Dental team members will then be guided through all the components of the Strong Teeth intervention. To ensure fidelity of the Strong Teeth intervention, dental team members will discuss the practicalities of delivering the intervention in their practice and agree upon a consistent approach to its delivery. Delivery will be reinforced with role play scenarios. An Oral-B representative (Professional Oral Health Territory Manager) will attend the training and provide a short tutorial on how to instruct parents to use the Oral-B electric rechargeable toothbrush with their child. During the study, a study team Dental Nurse (JP) will visit each practice and provide further training, role play and support to maximise the consistency of the Strong Teeth intervention.

#### Delivery of the Strong Teeth intervention

We will recruit dental teams from 4 to 8 dental practices who will deliver the Strong Teeth intervention as part of the child’s dental check-up and/or at a subsequent visit/s. Each dental team member delivering the Strong Teeth intervention will attend the training outlined above. In addition, parents will receive a toothbrush and guidance on how to use it. For children 0–2 years old, this will be a manual Oral-B toothbrush; for children 3–5 years old, a rechargeable Oral-B electric toothbrush will be provided.

#### Data analysis

The acceptability and feasibility of delivering the Strong Teeth intervention by the dental team will be explored in two ways. First, after delivering each intervention, dental team members will complete a semi-structured diary exploring how the visit went, what oral health barriers were identified, and what Strong Teeth resources were used. Second, having fully completed delivery of the Strong Teeth intervention for all the parents recruited, individual qualitative interviews and/or focus groups with the wider dental practice team will be undertaken. Interviews will be audio recorded, transcribed verbatim, and managed in NVivo. Data will be analysed using framework analysis guided by Ayala and Elder [29] recommendations and the Sekhon, Cartwright [30] theoretical framework of acceptability. This will be coded independently by two researchers, who will then compare codes and resolve any disagreements by discussion [31, 32].

Data regarding progression criteria (see Table 1), including recruitment and retention rates will be used to inform the decision to progress to a definitive trial, with the sample characteristics and overall recruitment and retention data being critical to the trial design.

### Acceptability of the Strong Teeth intervention for parents of children aged 0-5 years old and other outcomes measures

#### Sample size

Twenty parents of children 0–2 years old, and 20 parents of children 3–5 years old will be recruited to the study. The sample size has been derived to satisfy the best practice recommendations of Lancaster, Dodd [33] requiring at least 30 participants and will provide a 95% confidence interval of (74%, 96%) for a minimum anticipated retention rate of 85%. The data from the current feasibility study will inform and modify the sample size calculation for the subsequent definitive trial, although accepting the design (probably involving less home visits), primary outcome (dental decay) and follow-up (3 years) may differ.

Inclusion criteria:

- Children 0–5 years old about to visit their general dental practice for a dental check-up
- Children attending a general dental practice where the dental team is trained to deliver the Strong Teeth intervention

Exclusion criteria:

- Only one sibling can be recruited per household
- A parent must be present at the baseline home visit to ensure valid consent

Purposive sampling of parents and children will be undertaken to ensure the sample includes participants from different ethnic groups, living in areas of varying levels of deprivation, and with differing severities of dental decay. However, due to resource restraints, only parents who can understand intervention sessions delivered in English will be included.

### Acceptability to parents/children of the Strong Teeth intervention

The outcome measures and the measurement schedule will be captured through structured questionnaires at baseline, as well as 2 weeks and 2–3 months after the intervention. In addition, qualitative interviews will take place in the parental home at around 3 months after the intervention. An analytical approach using NVivo and theoretical framework analysis will be undertaken, similar to that described for dental teams above.

### Mechanism of action of the Strong Teeth intervention

Qualitative and quantitative data will be used to explore intervention mechanisms with questionnaires and interview topic guides being explicitly developed including questions mapped onto the Theoretical Domains Framework [31], considerate of wider family and community context, as tested and refined through our previous work [11, 12, 17, 21]. The intervention mechanism (i.e. what are the active ingredients within the intervention, and how they are exerting their effect) will be evaluated, and our generic intervention logic model refined [11].

### Adoption and maintenance of appropriate oral health behaviours

**Fig. 1:**
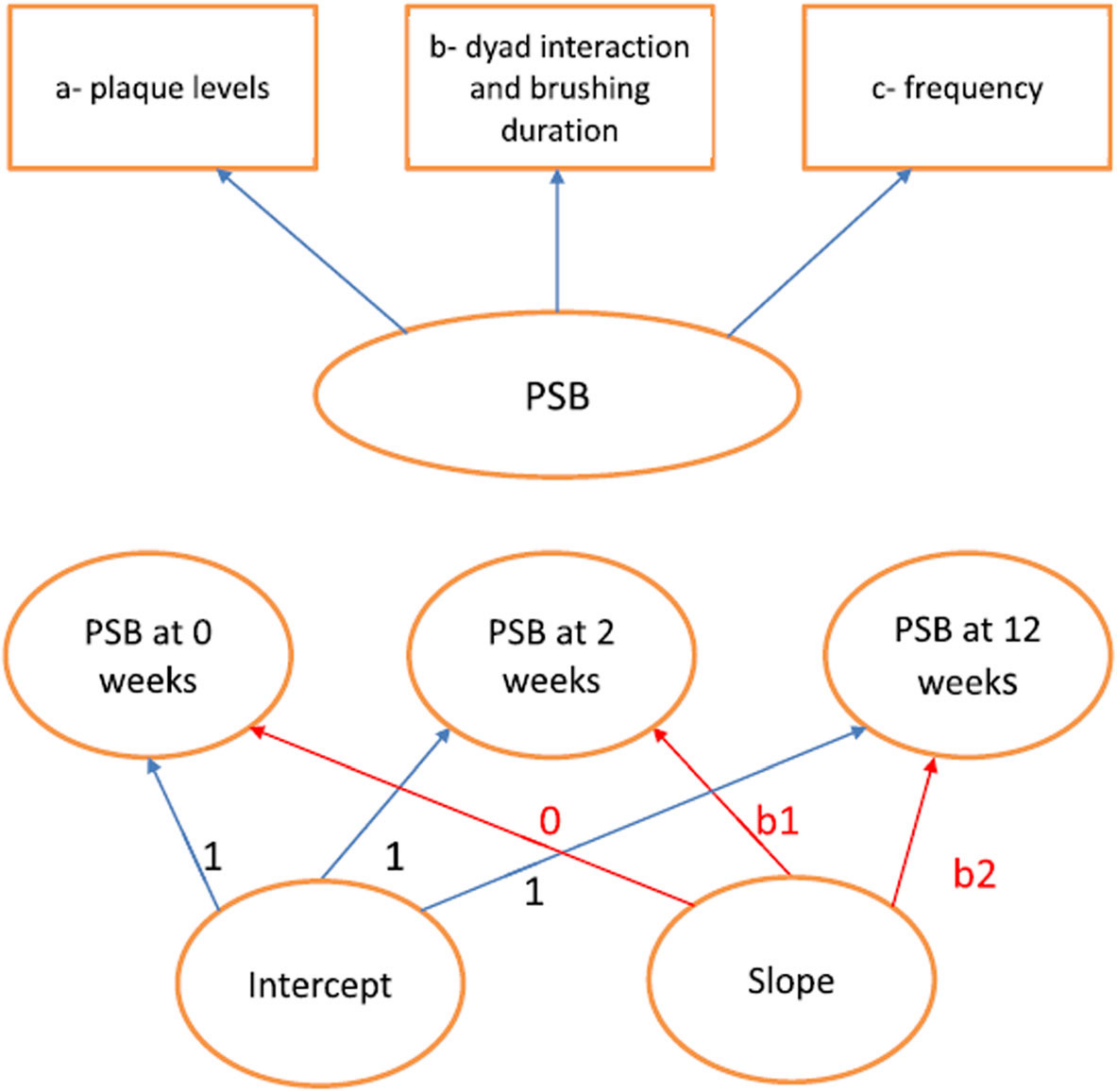
The measurement (top model) and growth (bottom model) models for the three proxy objective measures of parental supervised toothbrushing (PSB). Published with permission from Eskyte et al. [12]

Changes in self-report and objective measures of PSB behaviours will be collated. The adoption and maintenance of good oral health behaviours will be measured against national guidance—for example, parental supervised toothbrushing undertaken twice a day with the appropriate amount and strength of fluoride toothpaste [3]. The validity of parent/child reports of PSB behaviours will be compared with three proxy objective measures (1–3, listed in the “Design/methods” section). We will formulate a preliminary measurement model and calculate factor loadings. Factor loadings will be available from the measurement model. By generating a standardised model where the variance of each objective measure is scaled to unity, the associate standardised factor loadings will effectively rank the measures according to the strength of their contributions to PSB. These will be taken as the quantitative assessment for each measure. The same model was used for our HABIT early-phase study and can be seen in Fig. 1 [12]. Other measures of toothbrushing behaviour (such as duration of brushing, amount and strength of fluoride toothpaste used and spitting out toothpaste residue after brushing) will be considered for inclusion in the model.

The dietary data collected at baseline, 2 weeks and 2–3 months will allow changes in dietary behaviour to be evaluated with respect to the frequency of sugary foods and drinks consumed by children. This quantitative dietary data will be used in conjunction with qualitative findings.

### Impact of an Oral-B electric rechargeable toothbrush for children aged 3-5 years old

**Fig. 2:**
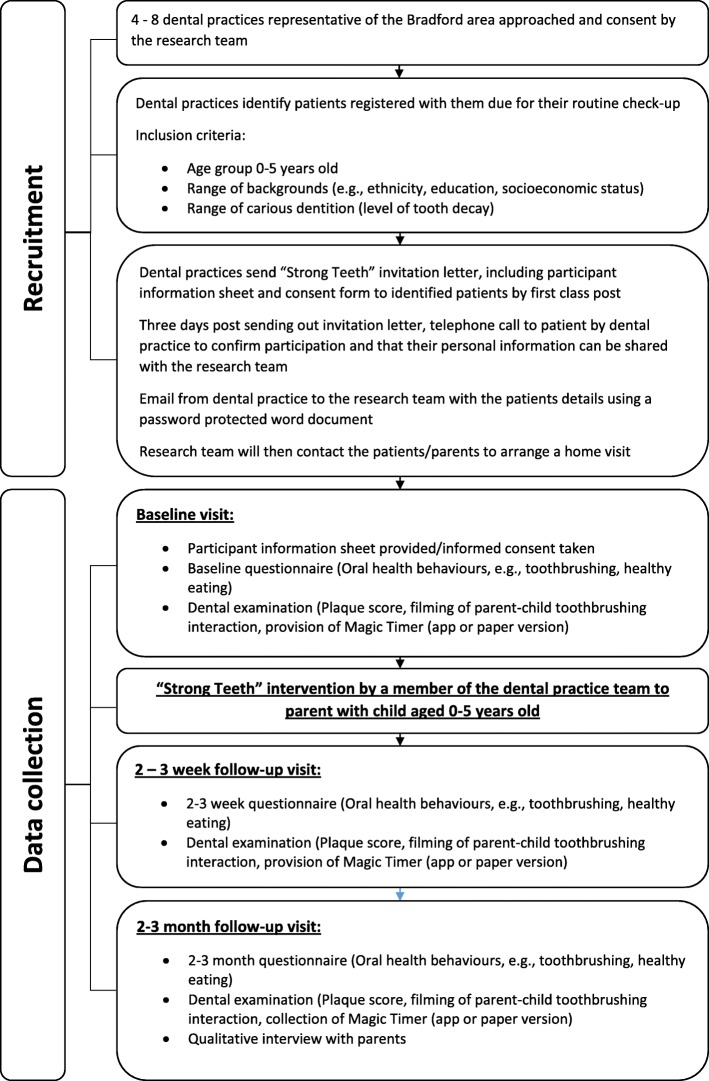
A detailed flowchart of the recruitment and data collection process

The impact of providing children aged 3–5 years old (*n* = 20) with an Oral-B electric rechargeable toothbrush will be evaluated. This will include assessing the acceptability of the electric toothbrush to children and parents. Furthermore, toothbrushing behaviours (frequency of toothbrushing, duration, amount and strength of fluoride toothpaste and spitting out toothpaste residue after brushing) will be explored in the home setting during data collection visits (at 2 weeks and 2–3 months post intervention) and with parents who agree to participate in the qualitative interviews (please see Fig. 2 for a detailed flowchart of the recruitment and data collection process).

## Discussion

This early phase study is designed to evaluate the Strong Teeth complex oral health intervention and inform the design of a definitive study to explore the impact of the intervention on dental caries in children. It will provide invaluable information regarding the acceptability, feasibility and impact of the intervention on both dental teams and parents of children aged 0–5 years old. Specifically, it will describe the capabilities and skills of dental teams and outline what training and support is needed for the successful delivering of the Strong Teeth intervention in a general dental practice setting. It will provide deeper insight into the internal (e.g. motivation) and external (e.g. cultural, societal, interactional, contextual) factors underlying parental oral health behaviours. Furthermore, the study will evaluate whether and how the Strong Teeth intervention shapes oral health behaviour changes and characterise the impact of providing children aged 3–5 years old with an Oral-B electric rechargeable toothbrush.

In conjunction with our HABIT early phase study exploring the feasibility and acceptability of an oral health intervention delivered by health visitors to parents of children aged 9-12 months old in the UK [12]; this study will continue the important work in addressing the lack of objective measures of PSB adoption. Whilst there are robust measures of dental caries, these require long-term follow-up (a minimum of 3 years) and are consequently more expensive and at high risk of attrition. Whilst short-term parental-self reports of PSB exist, these are at high risk of social desirability bias [34]. The size of this bias and the lack of objective measures that robustly characterise PSB behaviour is a key evidence gap that will be further addressed in this study. Whilst our earlier HABIT study focused on children aged 9–15 months, this study will examine the acceptability, feasibility and utility of these measures in older children aged 0–5 years old.

This study has learnt from the findings from our HABIT study early-phase evaluation and informed the progression criteria outlined in Table 1. The first home visit now combines both consent and baseline data collection. Furthermore, general dental practices will be supported throughout the study by regular visits from a member of the study team who is a dental nurse. She will provide weekly support to each practice to maintain their enthusiasm and focus on the study. Specifically, she will help support each practice with parent recruitment, consistency in the delivery of the Strong Teeth intervention and administration of study paperwork and governance. The benefits of this role will be generalisable to other research in primary dental care.

In terms of participant comfort, the study does not seek to reveal any sensitive issues, and it is not anticipated that the participants will feel distressed during the course of the research. However, some parents may find the discussions on current toothbrushing habits difficult or embarrassing. In order to support such parents, any oral health questions or concerns the parents may have will be answered either by their dental team at the Strong Teeth intervention visit or by the research team at the end of the study.

Parents’ participation in the study requires them to be involved in the research activities over a 2–3-month period. This necessitates that parents feel comfortable with the research team and the data collection methods. To maintain progress, enthusiasm and momentum of the study, home visits will be organised when most convenient to parents and their time and participation rewarded with a £10 Love2Shop voucher after each home-based data collection visit in compliance with the NHS Health Research Authority “Payments and Incentives in Research” ethics guidance.

Dental teams will be funded by the National Institute for Health Research (NIHR) Clinical Research Network, Yorkshire and Humber (NIHR CRN), and Procter and Gamble Company for their participation in the study, in recognition of the research responsibilities and additional activities. The study findings will be widely disseminated via academic, professional and public venues. Research findings will be published in a peer-reviewed health care journal and as conference abstracts and presentations. In terms of data distribution to professionals, an event for dental teams, public health professionals and commissioners will be organised at the end of the project, which will provide a platform to engage in further discussion. A wider programme of dissemination will also involve parents and the public. The findings will be disseminated back to the group of participants in a lay report and a video vignette that will be developed together with community members, who will advise on the most appropriate method of dissemination to the local community.

## Additional file

Additional file 1: Appendix 1 Structured interview guide for data collection from Parents at the first three meetings. Appendix 2 Duration of parent/child (dyad) interaction during toothbrushing. (ZIP 159 kb)

## Data Availability

As this is a protocol paper, no data is available, as of yet.

## References

[1] Department of Health (2012). Healthy lives, healthy people: improving outcomes and supporting transparency.

[2] Public Health England. National Dental Epidemiology Programme for England: oral health survey of five-year-old children 2017. A report on the inequalities found in prevalence and severity of dental decay; 2018.

[3] Public Health England (2017). Delivering Better Oral Health: an evidence-based toolkit for prevention.

[4] NICE (2014). Oral health: approaches for local authorities and their partners to improve the oral health of their communities.

[5] Hall-Scullin E, Goldthorpe J, Milsom K, Tickle M (2015). A qualitative study of the views of adolescents on their caries risk and prevention behaviours. BMC Oral Health.

[6] Hall-Scullin E., Whitehead H., Milsom K., Tickle M., Su T.-L., Walsh T. (2017). Longitudinal Study of Caries Development from Childhood to Adolescence. Journal of Dental Research.

[7] Broadbent JM, Thomson WM, Boyens JV, Poulton R (2011). Dental plaque and oral health during the first 32 years of life. J Am Dent Assoc..

[8] Aljafari AK, Scambler S, Gallagher JE, Hosey MT (2014). Parental views on delivering preventive advice to children referred for treatment of dental caries under general anaesthesia: a qualitative investigation. Community Dent Health.

[9] Aljafari AK, Gallagher JE, Hosey MT (2015). Failure on all fronts: general dental practitioners’ views on promoting oral health in high caries risk children--a qualitative study. BMC Oral Health..

[10] Ogretme MS, AbualSaoud D, Hosey MT (2016). What preventive care do sedated children with caries referred to specialist services need?. Br Dent J.

[11] Gray-Burrows K, Day PF, Marshman Z, Aliakbari E, Prady SL, McEachan RRC (2016). Using intervention mapping to develop a home-based parental supervised toothbrushing intervention for young children. Implement Sci..

[12] Eskyte I, Gray-Burrows K, Owen J, Sykes-Muskett B, Zoltie T, Gill S (2018). HABIT—an early phase study to explore an oral health intervention delivered by health visitors to parents with young children aged 9–12 months: study protocol. Pilot and feasibility Stud.

[13] Primary Care Domain ND (2018). NHS Dental Statistics, England.

[14] NICE. Dental checks: intervals between oral health reviews. 2004. http://nice.org.uk/guidance/cg19. 31869036

[15] Tickle M, O'Neill C, Donaldson M, Birch S, Noble S, Killough S (2016). A randomised controlled trial to measure the effects and costs of a dental caries prevention regime for young children attending primary care dental services: the Northern Ireland Caries Prevention In Practice (NIC-PIP) trial. Health Technol Assess..

[16] Craig P, Dieppe P, Macintyre S, Michie S, Nazareth I, Petticrew M (2008). Developing and evaluating complex interventions: the new Medical Research Council guidance. Bmj.

[17] Gray-Burrows KA, Owen J, Day PF (2017). Learning from good practice: a review of current oral health promotion materials for parents of young children. Bdj..

[18] Health and Social Care Information Centre. Child Dental Health Survey 2013, England, Wales and Northern Ireland [NS]. http://www.hscic.gov.uk/catalogue/PUB17137: 2015.

[19] Cade JE, Frear L, Greenwood DC (2006). Assessment of diet in young children with an emphasis on fruit and vegetable intake: using CADET--Child and Diet Evaluation Tool. Public Health Nutr..

[20] Greene JC, Vermillion JR (1964). The Simplified Oral Hygiene Index. J Am Dent Assoc (1939).

[21] Marshman Z, Ahern SM, McEachan RRC, Rogers HJ, Gray-Burrows KA, Day PF (2016). Parents’ experiences of toothbrushing with children: a qualitative study. JDR Clin Trans Res.

[22] Collett BR, Huebner CE, Seminario AL, Wallace E, Gray KE, Speltz ML. Observed child and parent toothbrushing behaviors and child oral health. Int J Paediatr Dent. 2015. 10.1111/ipd.12175 PMC5828507 26148197

[23] Zeedyk MS, Longbottom C, Pitts NB (2005). Tooth-brushing practices of parents and toddlers: a study of home-based videotaped sessions. Caries Res.

[24] Martin M, Rosales G, Sandoval A, Lee H, Pugach O, Avenetti D (2019). What really happens in the home: a comparison of parent-reported and observed tooth brushing behaviors for young children. BMC Oral Health.

[25] Public Health England. National Dental Epidemiology Programme for England: oral health survey of five-year-old children 2015 A report on the prevalence and severity of dental decay. 2016.

[26] Pine CM, Pitts NB, Nugent ZJ (1997). British Association for the Study of Community Dentistry (BASCD) guidance on the statistical aspects of training and calibration of examiners for surveys of child dental health. A BASCD coordinated dental epidemiology programme quality standard. Community Dental Health..

[27] Pitts NB, Evans DJ, Pine CM (1997). British Association for the Study of Community Dentistry (BASCD) diagnostic criteria for caries prevalence surveys-1996/97. Community Dental Health..

[28] Lally P, Wardle J, Gardner B (2011). Experiences of habit formation: a qualitative study. Psychol Health Med.

[29] Ayala GX, Elder JP (2011). Qualitative methods to ensure acceptability of behavioral and social interventions to the target population. J Public Health Dent.

[30] Sekhon M, Cartwright M, Francis JJ (2017). Acceptability of healthcare interventions: an overview of reviews and development of a theoretical framework. BMC Health Serv Res.

[31] Cane J, O'Connor D, Michie S (2012). Validation of the theoretical domains framework for use in behaviour change and implementation research. Implementation science: IS..

[32] Ritchie JL, Spencer L, BAR B (1994). Qualitative data analysis for applied policy research. Analyzing Qualitative Data.

[33] Lancaster GA, Dodd S, Williamson PR (2004). Design and analysis of pilot studies: recommendations for good practice. J Eval Clin Pract.

[34] Sanzone LA, Lee JY, Divaris K, DeWalt DA, Baker AD, Vann WF (2013). A cross sectional study examining social desirability bias in caregiver reporting of children’s oral health behaviors. BMC Oral Health..

