# Supplementary material for: “Strong Teeth”—a study protocol for an early-phase feasibility trial of a complex oral health intervention delivered by dental teams to parents of young children
Source: Pilot Feasibility Stud. 2019 Aug 13;5:100. doi: 10.1186/s40814-019-0483-9 (PMC6691530; doi:10.1186/s40814-019-0483-9)
Supplement: Supplementary file 1 — Appendix 1 Structured interview guide for data collection from Parents at the first three meetings. Appendix 2 Duration of parent/child (dyad) interaction during toothbrushing. (ZIP 159 kb) [file 40814_2019_483_MOESM1_ESM.zip › New folder/Appendix 1.docx]

Appendix 1

|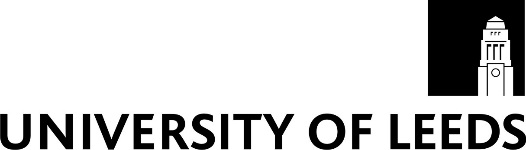

Structured interview guide for data collection from Parents at the first three meetings^[[1]](#endnote-1)^

**“Strong Teeth” programme: A study to explore the acceptability and impact of an oral health education programme delivered by dental teams to parents of young children**

Study Number: 248833, Version: 1, Date: 17-07-18

QUESTIONNAIRE 1 (BASELINE)

Participant study ID: ………………………………………………………………

Date completing the questionnaire: ……………………………………….

Researcher administering the questionnaire: …………………………

1. **Basic demographic and family questions**
   1. What is your date of birth?

Day Month Year

- 1. What country were you born in?

| 1 | England | 5 | Northern Ireland |
| --- | --- | --- | --- |
| 2 | Pakistan | 6 | Scotland |
| 3 | Bangladesh | 7 | Wales |
| 4 | India | 8 | Republic of Ireland |
| 9 | Other (please add) | | |

- 1. How many people live in your household, including yourself?
  2. How many children live in your household:

What are their age/s?…………………………………………………………………………………………..

- 1. How many children do you have?

What are their age/s? ………………………………………………………………………………………….

- 1. In which country have you obtained your highest educational qualification?

| 1 | England | 4 | India |
| --- | --- | --- | --- |
| 2 | Pakistan | 5 | Elsewhere |
| 3 | Bangladesh | 0 | No qualifications |

- 1. What is the highest educational level you have completed? *(Researcher to ask as an open question and populate one of the boxes as appropriate)*

| 1 | Less than 5 GCSEs (A*-C), CSE or O-levels | 11 | AS or A level, International Baccalaureate or BTEC |
| --- | --- | --- | --- |
| 2 | 5 or more GCSEs (A*-C), SCE or O-Levels | 12 | NVQ 4/5 |
| 3 | GNVQ foundation level | 13 | Higher National Certificate/ Higher National Diploma, Higher Education Diploma |
| 4 | NVQ1 | 14 | Foundation degree |
| 5 | GNVQ intermediate | 15 | Bachelor’s degree 3-5 years |
| 6 | NVQ2 | 16 | Master’s degree (taught/research) or Postgraduate qualification, Doctorate/ PhD |
| 7 | Young apprenticeship | 17 | Other: |
| 8 | NVQ3 |  |  |
| 9 | Advanced NVQ | 0 | None |
| 10 | GNVQ Advanced apprenticeship | 999 | Don’t know |

- 1. Are you currently employed?

| - *1.* Yes (go to 1.10) | - *2.* No (go to 1.9) |
| --- | --- |

- 1. If you are not currently employed, have you worked before?

| - *1.* Yes | - *2.* No |
| --- | --- |

- 1. Which of these best describes the sort of work you do/ did? *(Researcher to ask as an open question and populate* ***one*** *of the boxes as appropriate)*

| 1 | *Modern professional occupations*, such as: teacher – nurse - physiotherapist – social worker – welfare officer – artist– musician – police officer (sergeant or above) – software designer |
| --- | --- |
| 2 | *Clerical and intermediate occupations*, such as: secretary – personal assistant – clerical worker – office clerk – call centre - agent – nursing auxiliary – nursery nurse |
| 3 | *Senior managers or administrators* (usually responsible for planning, organising and co-ordinating work, and for finance), such as: finance manager – chief executive |
| 4 | *Technical and craft occupations*, such as: motor mechanic - fitter – inspector – plumber – printer – tool maker – electrician – gardener – train driver |
| 5 | *Semi-routine manual and service occupations*, such as: postal worker – machine operative – security guard – caretaker - farm worker – catering assistant – receptionist – sales - assistant |
| 6 | *Routine manual and service occupations*, such as: HGV driver – van driver – cleaner – porter – packer – sewing machinist – messenger – labourer – waiter/waitress – bar staff |
| 7 | *Middle or junior managers*, such as: office manager – retail manager – bank manager – restaurant manager – warehouse manager – publican |
| 8 | *Traditional professional occupations*, such as: accountant - solicitor – medical practitioner – scientist – civil/mechanical engineer |
| 9 | Other:  ……………………………………………………………………………………………………………………. |

1. **Family finances – for these two questions, the questionnaire is self-completed – please hand the paper to the participants – they are free not to answer if they do not want to**
   1. How well would you say you (and your partner) are managing financially these days?

| 1 | Living comfortably | 4 | Finding it quite difficult |
| --- | --- | --- | --- |
| 2 | Doing alright | 5 | Finding it very difficult |
| 3 | Just about getting by |  |  |
|  |  |  |  |
| 999 | Do not wish to answer | 0 | Don’t know |

- 1. We would like to know about the total income of your household. The total income of your household includes income from all jobs (full and part time), all tax credits, all benefits, and all other sources and earnings after tax when all income is added together. *Tick appropriate box*

| Group | **Weekly** | **Monthly** | **Yearly** |
| --- | --- | --- | --- |
| **1** | Less than £310 | Less than £1,304 | Less than £16,100 |
| **2** | £310 - £409 | £1,304-£1,769 | £16,100-£21,249 |
| **3** | £410 - £539 | £1,770-£2,329 | £21,250-£27,999 |
| **4** | £540-£739 | £2,330-£3,199 | £28,000-£38,399 |
| **5** | More than £739 | More than £3,199 | More than £38,399 |
|  |  |  |  |
| 999 | Do not wish to answer | | |

1. **Child’s health**
   1. What is your child’s date of birth?

Day Month Year

- 1. In your opinion, is your child fit and healthy? 1-Yes/ 2-No
  2. Do they see a paediatrician? 1-Yes/ 2-No

*If yes*, for what reason? ……………………………………………………………………………………………….

- 1. Is your child allergic to anything, including food dyes? 1-Yes/ 2-No

*If yes,* what?………………………………………………………………………………………………………………….

- 1. As far as you are aware, is your child meeting all their developmental milestones? 1-Yes/ 2-No

1. **Child’s diet**
   1. How often do you give your child these different foods and drinks? *(Researcher to ask as an open question and populate* ***one*** *of the boxes as appropriate)*

|  | *9* | *8* | *7* | *6* | *5* | *4* | *3* | *2* | *1* | *0* |
| --- | --- | --- | --- | --- | --- | --- | --- | --- | --- | --- |
|  | **6+ times a day** | **4-5 times per day** | **2-3 times per day** | **Once per day** | **5-6 times per week** | **2-4 times per week** | **Once per week** | **1-3 times per month** | **Less than one a month** | **Never** |
| Fresh fruit* |  |  |  |  |  |  |  |  |  |  |
| Vegetables (cooked or raw, root veg, salads, fresh or frozen) |  |  |  |  |  |  |  |  |  |  |
| Fresh meats, poultry and fish |  |  |  |  |  |  |  |  |  |  |
| Processed meat and fish products |  |  |  |  |  |  |  |  |  |  |
| Chips, roast or potato shapes |  |  |  |  |  |  |  |  |  |  |
| Crisps, or savoury snacks |  |  |  |  |  |  |  |  |  |  |
| Savoury commercial foods (including dried, jars and tinned varieties) |  |  |  |  |  |  |  |  |  |  |
| Cheese |  |  |  |  |  |  |  |  |  |  |
|  | *9* | *8* | *7* | *6* | *5* | *4* | *3* | *2* | *1* | *0* |
|  | **6+ times a day** | **4-5 times per day** | **2-3 times per day** | **Once per day** | **5-6 times per week** | **2-4 times per week** | **Once per week** | **1-3 times per month** | **Less than one a month** | **Never** |
| Potatoes, pasta, rice |  |  |  |  |  |  |  |  |  |  |
| Beans or pulses |  |  |  |  |  |  |  |  |  |  |
| Bread |  |  |  |  |  |  |  |  |  |  |
| Sweets, chocolate |  |  |  |  |  |  |  |  |  |  |
| Cakes, scones, sweet pies or pastries |  |  |  |  |  |  |  |  |  |  |
| Breakfast cereals |  |  |  |  |  |  |  |  |  |  |
| Ice cream |  |  |  |  |  |  |  |  |  |  |
| Savoury children’s food – commercially produced |  |  |  |  |  |  |  |  |  |  |
| Sweet children’s food – commercially produced |  |  |  |  |  |  |  |  |  |  |
| Biscuits |  |  |  |  |  |  |  |  |  |  |
| Fruit juice or children’s fruit juice (NOT squash) |  |  |  |  |  |  |  |  |  |  |
| Soft/ fizzy drinks or squashes (sugared) |  |  |  |  |  |  |  |  |  |  |
| Soft/ fizzy drinks or squashes (sugar free) |  |  |  |  |  |  |  |  |  |  |
| Water |  |  |  |  |  |  |  |  |  |  |
| Milk (cows, breast or formula) |  |  |  |  |  |  |  |  |  |  |

*What fresh fruit do you usually give to your child? ………………………………………………………………

……………………………………….……………………………………………………..………………………………………………...……………………………………………………………………………………………………………………………………………..

4.2 Are there any differences on weekdays vs weekend days? 1=Yes/ 2=No

*If yes,* please explain ………………………………………………………………………………………………………………

……………………………………………………………………………………………………………………………………………

4.3 Are there any differences on different seasons/times of the year? 1=Yes/ 2=No

*If yes,* please explain ……………………………………………………………………………………………………………..

....................................................................................................................................................

……………………………………………………………………………………………………………………………………………..

**Drinks**

- 1. What does your child drink from (e.g. bottle, beaker)? …………………………………..

………………………………………………………………………………………………………………………………….

- 1. What drinks does your child have in this bottle/beaker?.................................

………………………………………………………………………………………………………………………………….

- 1. Does your child have access to these drinks throughout the day/night, or do they use it/them at mealtimes only?............................................................................

…………………………………………………………………………………………………………………………………

- 1. Does your child suck a dummy or their thumb? 1=Yes/ 2=No

If they suck a dummy, do you dip the dummy in anything, and if so what? ………………………………………………………………………………………………………………………

………………………………………………………………………………………………………………………………….

- 1. At bedtime, what is your child's routine? For example, does your child have a bedtime drink, and if so, what is in it? …………………..…………………………………………………

………………………………………………………………………………………………………………………………….

………………………………………………………………………………………………………………………………….

1. **Looking after your child’s teeth**
   1. How many teeth does your child have? …………………………..
   2. Have you started brushing your child’s teeth?
   - *1.* Yes
   - *2.* No

**The following questions relate to thoughts and feelings around toothbrushing, and are relevant whether toothbrushing has commenced or not. Parent may provide these answers without necessarily being asked each question.**

1. Please indicate you agreement with the following statements:

|  | Strongly disagree | Disagree | Neither agree nor disagree | Agree | Strongly agree |
| --- | --- | --- | --- | --- | --- |
| I have the necessary knowledge and skills to be able to brush my child’s teeth | 1 | 2 | 3 | 4 | 5 |
| I am confident in my ability to brush my child’s teeth | 1 | 2 | 3 | 4 | 5 |
| I can manage my child’s behaviours to allow me to brush their teeth | 1 | 2 | 3 | 4 | 5 |
| I intend to brush my child’s teeth | 1 | 2 | 3 | 4 | 5 |
| As a parent, it is my responsibility to brush my child’s teeth | 1 | 2 | 3 | 4 | 5 |
| I have enough time to brush my child’s teeth | 1 | 2 | 3 | 4 | 5 |
| My financial situation enables me to buy a toothbrush and toothpaste for my child | 1 | 2 | 3 | 4 | 5 |
| Most people important to me think that my child should have their teeth brushed | 1 | 2 | 3 | 4 | 5 |
| I receive appropriate support from those around me to help me brush my child’s teeth | 1 | 2 | 3 | 4 | 5 |
| Brushing my child’s teeth will prevent tooth decay | 1 | 2 | 3 | 4 | 5 |

1. Thinking of all the things you need to do to keep your child healthy, how important is brushing your child’s teeth? *(indicate on scale)*

Not important Very important

1 2 3 4 5

***If toothbrushing has not commenced then this is the end of the questionnaire.***

***The following questions relate to current tooth brushing practices where brushing HAS started. Parent may provide these answers without necessarily being asked each question.***

1. Can you describe how you brush your child’s teeth?

……………………………………………………………………………………………………………………………………………………………………………………………………………………………………………………………………………………………………………………………………………………………………………………………………………………………………………………………………………………………………………………………………………………

1. What kind of toothbrush do you use*? (Researcher to ask as an open question and populate one of the boxes as appropriate)*

| 1 | Regular child’s toothbrush |
| --- | --- |
| 2 | Finger toothbrush |
| 3 | Adult toothbrush |
| 4 | Electric toothbrush |

1. Do you use any other oral health products for your child (e.g. mouthwash, teething gel etc.)? 1=Yes/ 2=No

If yes, please state which

……………………………………………………………………………………………………………………………………………………………………………………………………………………………………………………………………………………………………………………………………………………………………………………………………………………………………………………………………………………………………………………………………………………

1. Who usually brushes your child’s teeth? *(Researcher to ask as an open question and populate one of the boxes as appropriate)*

- *1.* Your child
- *2.* You
- *3.* Another adult (identify who) ………………………………………….…………………………………………………………………..

| 4 | Mum and Dad | 5 | Child and Mum | 6 | Mum and other adult | 7 | Mum, Dad and other adult | 8 | Mum, Dad and child | 9 | Mum, other adult, and child |
| --- | --- | --- | --- | --- | --- | --- | --- | --- | --- | --- | --- |

*10.* other……………………………………………………………………………………………………………

1. Do you use toothpaste? 1-Yes/ 2-No (go to 16)
2. If you use toothpaste, at the end of brushing your child’s teeth, do you encourage your child to: *(Researcher to ask as an open question and populate* ***one*** *of the boxes as appropriate)*

| 1 | Rinse their mouth after brushing (e.g. water) |
| --- | --- |
| 2 | Just spit out the toothpaste residue |
| 3 | Wipe away the excess toothpaste |
| 4 | Eats and or swallows the toothpaste |

1. If you use toothpaste with your child, what is the name of the toothpaste you are currently using to brush your child’s teeth? *Please write the full name of toothpaste*:

(e.g. Colgate Total Care, Tesco Steps Toothpaste, Green People Organic Children Mandarin and Aloe Vera, Booths Fluoride Free, etc.).

…………………………………………………………………………………………………………………………………………….…………………………………………………………………………………………………………………………………………………………………………………………………………………………………………………..……..

1. If you use toothpaste, how much toothpaste do you use? *(Researcher will ask parent to describe amount used) …………………………………………………………………………………………………*

*…………………………………………………………………………………………………………………………………..*

1. How often do you brush your child’s teeth? *(Researcher to ask as an open question and populate one of the boxes as appropriate)*

| 1 | More than three times a day | 5 | Less than once a day |
| --- | --- | --- | --- |
| 2 | Three times a day | 6 | A few times a month |
| 3 | Twice a day | 7 | Occasionally, when my child is calm |
| 4 | Once a day | 0 | Never |

1. How would you describe the experience of brushing your child’s teeth?

……………………………………………………………………………………………………………………………………………………………………………………………………………………………………………………………………………………………………………………………………………………………………………………………………………………………………………………………………………………………………………………………………………………

…………………………………………………………………………………………………………………………………………………………………………………………………………………………………………………………………………

1. How do you feel when you brush your child’s teeth?

………………………………………………………………………………………………………………………………………………………………………………………………………………………………………………………………………………………………………………………………………………………………………………………………………………

………………………………………………………………………………………………………………………………………………………………………………………………………………………………………………………………………………………………………………………………………………………………………………………………………..…….

1. Do you ever forget to brush your child’s teeth?

- *1.* Yes
- *2.* No

1. What prompts you to brush your child’s teeth?

……………………………………………………………………………………………………………………………………………………………………………………………………………………………………………………………………………………………………………………………………………………………………………………………………………………………………………………………………………………………………………………………………………………………………………………………………………………………………………………………………………

***The following questions relate to initial tooth brushing practices, parent may provide these answers without necessarily asking each question.***

1. When did you start brushing your child’s teeth? *(Researcher to ask as an open question and populate one of the boxes as appropriate)*

| 1 | Under 3 months |
| --- | --- |
| 2 | Under 6 months |
| 3 | Under 9 months |
| 4 | Under 12 months |

1. In comparison to now – how did you initially carry out brushing your child’s teeth?

…………………………………………………………………………………………………………………………………………………………………………………………………………………………………………………………………………………………………………………………………………………………………………………………………………………………………………………………………………………………………………………………………………………………………………

1. In comparison to now – what method did you use (*we can explore type of toothbrush, type of toothpaste, how much, how often, why did they start brushing, who normally brushes)*?

…………………………………………………………………………………………………………………………………………………………………………………………………………………………………………………………………………………………………………………………………………………………………………………………………………………………………………………………………………………………………………………………………………………………………………

1. Dental questions Adapted from the 2013 Dental Health Survey of Children and Young People. Questionnaire for parents and guardians. Health and Social Care Information Centre

   Demographic, background and diet questions adapted from the Born in Bradford Better Start (2017) Questionnaire for Pregnant Women

   Appendix 2


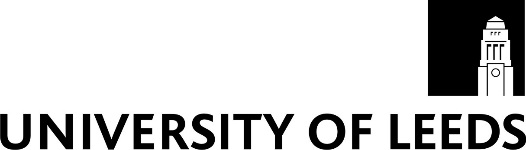


   Duration of parent/child (dyad) interaction during toothbrushing

   **“Strong Teeth” programme: A study to explore the acceptability and impact of an oral health education programme delivered by dental teams to parents of young children**

   Study Number: 248833, Version: 1, Date: 17-07-18

   The guide will be used to analyse the duration of and parent/child (dyad) interaction during toothbrushing. The researcher will film the dyad toothbrushing and this will be subsequently evaluated by the research team using an established parental supervised toothbrushing (PSB) index.

   Parents will be asked to ‘brush your child’s teeth as you would normally do’ and will be allowed to determine how long to brush, and whether brushing will be done by a child, parent, or combination.

   | **Parent Items** | **Yes** | **No** | **N/A** |
   | --- | --- | --- | --- |
   | Offered one or more appropriate choices |  |  |  |
   | Physically scaffolded the interaction |  |  |  |
   | Prompted child to open his/her mouth wide |  |  |  |
   | One or more positive messages to motivate (e.g., Your teeth will be so clean) |  |  |  |
   | Laughed or smiled during toothbrushing |  |  |  |
   | Used bribery to resolve problem behaviour |  |  |  |
   | 3 or more unlabelled praise statements related to toothbrushing |  |  |  |
   | Used distraction to help child to brush teeth (e.g., singing a toothbrushing song) |  |  |  |
   | Yelled at child or raised voice |  |  |  |
   | 1 or more labelled praise statements related to toothbrushing |  |  |  |
   | Made threats |  |  |  |
   | *Parental score:* |  |  |  |
   | **Child Items** |  |  |  |
   | Easily transitioned to toothbrushing |  |  |  |
   | Imitated parent’s demonstrations of toothbrushing |  |  |  |
   | Played with toothbrush, toothpaste, or water > 50% of the time |  |  |  |
   | Looked at parent’s face |  |  |  |
   | Focused attention on toothbrushing > 50% of the time |  |  |  |
   | One or more verbal descriptions of toothbrushing |  |  |  |
   | Sucked, bit, or chewed on toothbrush |  |  |  |
   | Initiated gentle, social physical contact with parent (e.g., sitting on parent’s lap without prompt) |  |  |  |
   | Gentle with parent and toothbrushing materials |  |  |  |
   | Asked questions related to toothbrushing |  |  |  |
   | “Bossy” towards parent |  |  |  |
   | Responded to parent’s cues > 50% of the time |  |  |  |
   | Showed any distress |  |  |  |
   | Comforted by parent’s response to distress |  |  |  |
   | Showed any refusal behaviour |  |  |  |
   | Whined, complained, or other mild verbal protest |  |  |  |
   | Yelled or screamed |  |  |  |
   | One or more major refusal behaviours |  |  |  |
   | *Child Score:* |  |  |  |

   Adapted from Collett, B. R. (2016) Observed child and parent toothbrushing behaviours and child oral health, International Journal of Paediatric Dentistry, 2016; 26: 184-192. [↑](#endnote-ref-1)
